# The sound of your lips: electrophysiological cross-modal interactions during hand-to-face and face-to-face speech perception

**DOI:** 10.3389/fpsyg.2014.00420

**Published:** 2014-05-13

**Authors:** Avril Treille, Coriandre Vilain, Marc Sato

**Affiliations:** CNRS, Département Parole and Cognition, Gipsa-Lab, UMR 5216, Grenoble UniversitéGrenoble, France

**Keywords:** audio-visual speech perception, audio-haptic speech perception, multisensory interactions, EEG, auditory evoked potentials

## Abstract

Recent magneto-encephalographic and electro-encephalographic studies provide evidence for cross-modal integration during audio-visual and audio-haptic speech perception, with speech gestures viewed or felt from manual tactile contact with the speaker’s face. Given the temporal precedence of the haptic and visual signals on the acoustic signal in these studies, the observed modulation of N1/P2 auditory evoked responses during bimodal compared to unimodal speech perception suggest that relevant and predictive visual and haptic cues may facilitate auditory speech processing. To further investigate this hypothesis, auditory evoked potentials were here compared during auditory-only, audio-visual and audio-haptic speech perception in live dyadic interactions between a listener and a speaker. In line with previous studies, auditory evoked potentials were attenuated and speeded up during both audio-haptic and audio-visual compared to auditory speech perception. Importantly, the observed latency and amplitude reduction did not significantly depend on the degree of visual and haptic recognition of the speech targets. Altogether, these results further demonstrate cross-modal interactions between the auditory, visual and haptic speech signals. Although they do not contradict the hypothesis that visual and haptic sensory inputs convey predictive information with respect to the incoming auditory speech input, these results suggest that, at least in live conversational interactions, systematic conclusions on sensory predictability in bimodal speech integration have to be taken with caution, with the extraction of predictive cues likely depending on the variability of the speech stimuli.

## INTRODUCTION

How information from different sensory modalities, such as sight, sound and touch, is combined to form a single coherent percept? As central to adaptive behavior, multisensory integration occurs in everyday life when natural events in the physical world have to be integrated from different sensory sources. It is an highly complex process known to depend on the temporal, spatial and causal relationships between the sensory signals, to take place at different timescales in several subcortical and cortical structures and to be mediated by both feedforward and backward neural projections. In addition to their coherence, the perceptual saliency and relevance of each sensory signal from the external environment, as well as their predictability and joint probability to occur, also act on the integration process and on the representational format at which the sensory modalities interface (for reviews, see Stein and Meredith, 1993; Stein, 2012).

Audio-visual speech perception is a special case of multisensory processing that interfaces with the linguistic system. Although one can extract phonetic features from the acoustic signal alone, adding visual speech information from the speaker’s face is known to improve speech intelligibility in case of a degraded acoustic signal (Sumby and Pollack, 1954; Benoît et al., 1994; Schwartz et al., 2004), to facilitate the understanding of a semantically complex statement (Reisberg et al., 1987) or a foreign language (Navarra and Soto-Faraco, 2005), and to benefit hearing-impaired listeners (Grant et al., 1998). Conversely, in laboratory settings, adding incongruent visual speech information may interfere with auditory speech perception and even create an illusory percept (McGurk and MacDonald, 1976). Finally, as in other cases of bimodal integration, audio-visual speech integration depends on the perceptual saliency of both the auditory (Green, 1998) and visual (Campbell and Massaro, 1997) speech signals, as well as their spatial (Jones and Munhall, 1997) and temporal (van Wassenhove et al., 2003) relationships.

At the brain level, several magneto-encephalographic (MEG) and electro-encephalographic (EEG) studies demonstrate that visual speech input modulates auditory activity as early as 50–100 ms in the primary and secondary auditory cortices (Sams et al., 1991; Klucharev et al., 2003; Lebib et al., 2003; Besle et al., 2004; Hertrich et al., 2007; Winneke and Phillips, 2011). Importantly, it has been shown that both the latency and amplitude of auditory evoked responses (N1/P2, M100) are attenuated and speeded up during audio-visual compared to auditory-only speech perception (Klucharev et al., 2003; Besle et al., 2004; van Wassenhove et al., 2005; Stekelenburg and Vroomen, 2007; Arnal et al., 2009; Pilling, 2010; Vroomen and Stekelenburg, 2010; Baart et al., 2014; Treille et al., 2014). Moreover, N1/P2 latency facilitation also appears to be directly function of the visemic information, with the higher visual recognition of the syllable, the longer latency facilitation (van Wassenhove et al., 2005; Arnal et al., 2009). Since the visual speech signal preceded the acoustic speech signal by 10s or 100s of milliseconds in these studies, the observed speeding-up and amplitude suppression of auditory evoked potentials might both reflect non-speech specific temporal (Stekelenburg and Vroomen, 2007; Vroomen and Stekelenburg, 2010) and phonetic (van Wassenhove et al., 2005; Arnal et al., 2009) visual predictions of the incoming auditory syllable (for recent discussions, see Arnal and Giraud, 2012; van Wassenhove, 2013; Baart et al., 2014).

Interestingly, speech can be perceived not only by the ear and by the eye but also by the hand, with orofacial speech gestures felt and monitored from manual tactile contact with the speaker’s face. Past studies on the Tadoma method provide evidence for successful communication abilities in trained deaf-blind individuals through the haptic modality (Alcorn, 1932; Norton et al., 1977). A few behavioral studies also demonstrate the influence of tactile information on auditory speech perception in untrained individuals without sensory impairment, especially in case of noisy or ambiguous acoustic signals (Fowler and Dekle, 1991; Gick et al., 2008; Sato et al., 2010). In a recent EEG study (Treille et al., 2014), electrophysiological evidence of cross-modal interactions was found during both audio-visual and audio-haptic speech perception, through the course of live dyadic interactions between a listener and a speaker. In this study, participants were seated at arm’s length from an experimenter and they were instructed to manually categorize /pa/ or /ta/ syllables presented auditorily, visually and/or haptically. In line with the above-mentioned EEG/MEG studies, N1 auditory evoked responses were attenuated and speeded up during live audio-visual speech perception. Crucially, haptic information was also found to speed up auditory speech processing as early as 100 ms. Given the temporal precedence of the dynamic configurations of the articulators on the auditory signal, as attested in a behavioral control experiment, the observed audio-haptic interactions in the listener’s brain raise the possibility that the brain use predictive temporal and/or phonetic relevant tactile information for auditory processing, despite less natural processing to extract relevant speech information from the haptic modality. From this possibility, however, a clear limit of this study comes from the use of a simple two-alternative forced-choice identification task between /pa/ and /ta/ syllables and an insufficient number of trials for reliable EEG analyses per syllable.

To further explore whether perceivers might integrate tactile information in auditory speech perception as they do with visual information, the present study aimed at replicating the observed bimodal interactions during live face-to-face and hand-to-face speech perception (Treille et al., 2014). As observed in previous studies on audio-visual speech perception (van Wassenhove et al., 2005; Arnal et al., 2009), we also specifically tested whether modulation of N1/P2 auditory evoked potentials during both audio-visual and audio-haptic speech perception might depend on the degree to which the haptic and visual signals predict the incoming auditory speech target. To this aim, the experimental procedure was adapted from the Tadoma method and similar to that previously used by Treille et al. (2014), except the use of a three-alternative forced-choice identification task between /pa/, /ta/, and /ka/ syllables and a sufficient number of trials for reliable EEG analyses per syllable. A gradient of visual and haptic recognition between the three syllables was first attested in a behavioral experiment, which was a requirement to assess visual and haptic predictability on the incoming auditory signal in a subsequent EEG experiment. In line with previous EEG studies on audio-visual speech integration (van Wassenhove et al., 2005; Arnal et al., 2009), we hypothesized that the higher visual and haptic recognition of the syllable, the stronger latency facilitation in the audio-visual and audio-haptic modalities.

## MATERIALS AND METHODS

### PARTICIPANTS

Sixteen healthy adults, native French speakers, participated in the study (eight females; mean age ± SD, 29 ± 8 years). All participants were right-handed, had normal or corrected-to-normal vision and reported no history of speaking, hearing or motor disorders. Written informed consent was obtained for all participants and they were compensated for the time spent in the study. The study was approved by the Grenoble University Ethical Committee.

### STIMULI

Based on a previous EEG study (van Wassenhove et al., 2005), /pa/, /ta/, and /ka/ syllables were selected in order to ensure precise acoustic onsets (thanks to the unvoiced stop bilabial /p/, alveolar /t/, and velar /k/ stop consonants) crucial for EEG analyses and, importantly, to ensure a gradient of visual and haptic recognition between these syllables (with notably the bilabial /p/ consonant known to be more visually salient than alveolar /t/ and velar /k/ consonants).

### EXPERIMENTAL PROCEDURE

The study consisted on one behavioral experiment immediately followed by one EEG experiment. The behavioral experiment was performed in order to ensure a gradient of visual and haptic recognition of /pa/, /ta/, and /ka/ syllables. Importantly, since individual syllable onsets of the experimenter’s productions were used as acoustical triggers for EEG analyses, the visual and haptic modalities of presentation were not included in the EEG experiment. In both experiments, Presentation software (Neurobehavioral Systems, Albany, CA, USA) was used to control the visual stimuli for the experimenter, the audio stimuli (beep) for the participant and to record key responses. In addition, all experimenter productions were recorded for off-line analyses in the EEG experiment.

#### Behavioral experiment

**FIGURE 1:**
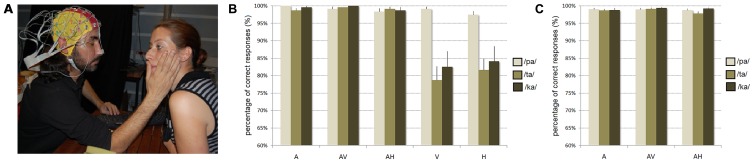
**(A)** Experimental design used in the audio-haptic (AH) modality. In the haptic (H) and AH modalities, participants were asked to keep their eyes closed with their right hand placed on the experimenter’s face and to categorize with their left hand each perceived syllable. In the auditory modality (A), participants were instructed to keep their eyes closed while, in the visual (V) and audio-visual modality (AV), they were asked to also look at the experimenter’s face. The behavioral experiment included A, V, H, AV, AH modalities while the EEG experiment only included A, AV, and AH modalities. **(B,C)** Mean percentage of correct identification for /pa/, /ta/, and /ka/ syllables in each modality of presentation in the **(B)** behavioral and **(C)** EEG experiments. Error bars represent standard errors of the mean.

In a first behavioral experiment, participants were individually tested in a sound-proof room and were seated at arm’s length from a female experimenter (see **Figure 1A**).

They were told that they would be presented with /pa/, /ta/, or /ka/ syllables either auditorily, visually, audio-visually, haptically, or audio-haptically over the hand-face contact. In the auditory modality (A), participants were instructed to keep their eyes closed and to listen to each syllable overtly produced by the experimenter. In the audio-visual modality (AV), they were asked to also look at the experimenter’s face. In the audio-haptic modality (AH), they were asked to keep their eyes closed with their right hand placed on the experimenter’s face (the thumb placed lightly and vertically against the experimenter’s lips and the other fingers placed horizontally along the jaw line in order to help distinguishing both lip and jaw movements). This experimental procedure was adapted from the Tadoma method and similar to that previously used by Treille et al. (2014). Finally, the visual-only (V) and haptic-only (H) modalities were similar to the AV and AH modalities except that the experimenter silently produced each syllable.

The experimenter faced the participant and a computer screen placed behind the participant. On each trial, the computer screen specified the syllable to be produced. To this aim, the syllable was printed three times on the computer screen at 1 Hz, with the last display serving as the visual go-signal to produce the syllable. The inter-trial interval was 3 s. The experimenter previously practiced and learned to articulate each syllable in synchrony with the visual go-signal, with an initial neutral closed-mouth position and maintaining an even intonation, tempo and vocal intensity.

A three-alternative forced-choice identification task was used, with participants instructed to categorize each perceived syllable by pressing on one of three keys corresponding to /pa/, /ta/, or /ka/ on a computer keyboard with their left hand. A brief single audio beep was delivered 600 ms after the visual go-signal (expecting to occur in synchrony with the experimenter production) with the participants told to produce their responses only after this audio go-signal. This procedure was done in order to dissociate sensory/perceptual responses from motor responses on EEG data in the next experiment. As a consequence, no reaction-times were acquired and only response rate were considered in further analyses.

Every syllable (/pa/, /ta/, or /ka/) was presented 15 times in each modality (A, V, H, AV, AH) in a single randomized sequence for a total of 225 trials. The response key designation were counterbalanced across participants. Before the experiment, participants performed few practice trials in all modalities. They received no instructions concerning how to interpret visual and haptic information but they were asked to pay attention to both modalities during bimodal presentation.

#### EEG experiment

Because of no possible reliable acoustical triggers in the visual-only and haptic-only modalities, the EEG experiment only included three individual experimental sessions related to A, AV, and AH modalities of presentation. Except this difference and the number of trials, the experimental procedure was identical to that used in the behavioral experiment. In each session, every syllable (/pa/, /ta/, or /ka/) was presented 80 times in a randomized sequence for a total of 240 trials. The order of the modality of presentation and the response key designation were fully counterbalanced across participants. Because the experimental procedure was quite taxing, each experimental session was split into two blocks of around 6 min each, allowing short breaks for both the experimenter and the participants.

### EEG ACQUISITION

In the EEG experiment, EEG data were continuously recorded from 64 scalp electrodes (Electro-Cap International, INC., according to the international 10–20 system) using the Biosemi ActiveTwo AD-box EEG system operating at a sampling rate of 256 Hz. Two additional electrodes served as reference (common mode sense [CMS] active electrode) and ground (driven right leg [DRL] passive electrode). One other external reference electrode was at the top of the nose. The electro-oculogram measuring horizontal (HEOG) and vertical (VEOG) eye movements were recorded using electrodes at the outer canthus of each eye as well as above and below the right eye. Before the experiment, the impedance of all electrodes was adjusted to get low offset voltages and stable DC.

### DATA ANALYSES

#### Behavioral analyses

In both the behavioral and EEG experiments, the proportion of correct responses was individually determined for each participant, each syllable and each modality. Two-way repeated-measure ANOVAs were performed on these data with the modality (A, V, H, AV, AH in the behavioral experiment; A, AV, AH in the EEG experiment) and the syllable (/pa, /ta/, /ka/) as within-subjects variables.

#### Acoustical analyses

In the EEG experiment, acoustical analyses were performed on the experimenter’s recorded syllables in order to determine the individual syllable onsets serving as acoustical triggers for the EEG analyses. All acoustical analyses were performed using Praat software (Boersma and Weenink, 2013). First, an automatic procedure based on an intensity and duration algorithm detection roughly identified each syllable’s onset in the A, AV, and AH modalities (11520 utterances). For all syllables, these onsets were further manually and precisely determined, based on waveform and spectrogram information related to the acoustic characteristics of voiced stop consonants. Omissions and wrong productions were identified and removed from the analyses (less than 1%).

#### EEG analyses

EEG data were processed using the EEGLAB toolbox (Delorme and Makeig, 2004) running on Matlab (Mathworks, Natick, MA, USA). Since N1/P2 auditory evoked potentials have maximal response over central sites on the scalp (Scherg and Von Cramon, 1986; Näätänen and Picton, 1987), EEG data preprocessing and analyses were conducted on three central electrodes (C3, Cz, C4). These electrodes, covering left, middle, and right central sites, were also selected based on previous EEG studies on audio-visual speech perception (e.g., Klucharev et al., 2003; Besle et al., 2004; Pilling, 2010; Treille et al., 2014). EEG data were first re-referenced off-line to the nose recording and band-pass filtered using a two-way least-squares FIR filtering (1–20 Hz). Data were then segmented into epochs of 1000 ms (from -500 ms to +500 ms to the acoustic syllable onset, individually determined from the acoustical analyses), with the prestimulus baseline defined from -500 ms to -400 ms. Epochs with an amplitude change exceeding ±60 μV at any channel (including HEOG and VEOG channels) were rejected (on average, less than 10%).

For each participant and each modality, the peak latency of auditory N1 and P2 evoked responses were first determined on the EEG waveform averaged over all electrodes and syllables. For each syllable, two temporal windows were then defined on these peaks ±30 ms in order to individually calculate N1 and P2 amplitude and latency on the related average waveform of C3, Cz, C4 electrodes. Two-way repeated-measure ANOVAs were then performed on N1 and P2 amplitude and latency with the modality (A, AV, AH) and the syllable (/pa/, /ka/, /ta/) as within-subjects variables.

In order to confirm previous EEG/MEG studies demonstrating that P2 and M100 latency reduction in the audio-visual modality vary as a function of the visual recognition of the presented syllable (van Wassenhove et al., 2005; Arnal et al., 2009), additional Pearson’s correlation analyses were carried out. These correlation analyses were performed between the individual visual and haptic recognition scores of the three syllables in the behavioral experiment and the related latency facilitation and reduction amplitude observed in the AV and AH modalities in the EEG experiment (leading to 3 × 16 correlation points per measure and per modality). In addition to raw data, these analyses were also performed on individual *Z*-score normalized data, in order to take account of individual differences.

## RESULTS

For all the following analyses, the significance level was set at *p* = 0.05 and Greenhouse–Geisser corrected (for violation of the sphericity assumption) when appropriate. When required, *post hoc* analyses were conducted with Newman–Keuls tests.

### BEHAVIORAL ANALYSES

#### Behavioral experiment (see Figure 1B)

Overall, the mean proportion of correct responses was of 94%. The main effect of modality of presentation was significant [*F*(4,60) = 33.67, *p* < 0.001], with more correct responses in A, AV, and AH modalities than in V and H modalities (as shown by *post hoc* analyses, all *p*’s < 0.001). Significant differences were also observed between syllables [*F*(2,30) = 15.59, *p* < 0.001], with more correct responses for /pa/ than for /ta/ and /ka/ syllables (as shown by *post hoc* analyses, all *p*’s < 0.001). Finally, the interaction between the modality and the syllable was also reliable [*F*(8,120) = 7.39, *p* < 0.001]. While no significant differences were observed between syllables in A, AV, and AH modalities (with almost perfect identification for all syllables), more correct responses were observed for /pa/ than for /ta/ and /ka/ syllables in both V and H modalities (as shown by *post hoc* analyses, all *p*’s < 0.001). Altogether, these results thus demonstrate a near perfect identification of /pa/ in all modalities, but a lower accuracy for /ta/ and /ka/ syllables in V and H modalities.

#### EEG experiment (see Figure 1C)

In the EEG experiment, the mean proportion of correct responses was of 99%. No significant effect of the modality [*F*(2,30) = 1.72], syllable [*F*(2,30) = 1.34] or interaction [*F*(4,60) = 0.90] was observed, with a near perfect identification of all syllables in A, AV, and AH modalities.

### EEG ANALYSES

#### N1 amplitude (see Figures 2 and 3A-left)

**FIGURE 2:**
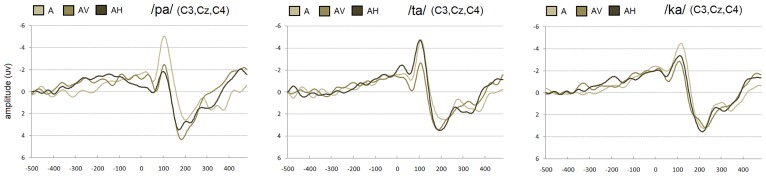
**Grand-average of auditory evoked potentials for /pa/, /ta/, and /ka/ syllables averaged over the left (C3), middle (Cz), and right (C4) central electrodes in the auditory, audio-visual, and audio-haptic modalities**.

**FIGURE 3:**
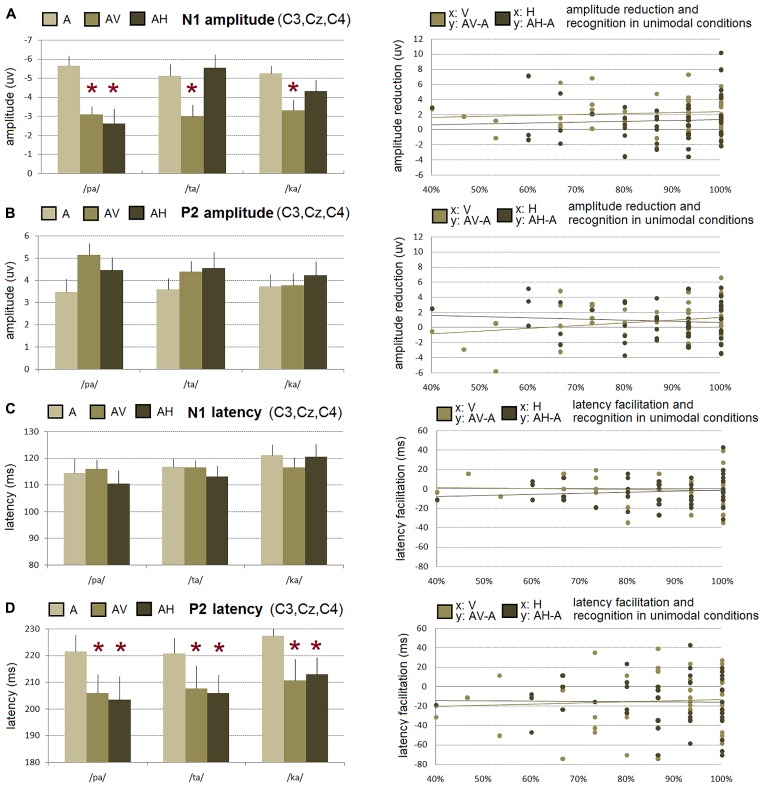
**Left.** Mean N1 **(A)** and P2 **(B)** amplitude and mean N1 **(C)** and P2 **(D)** latency for /pa/, /ta/, and /ka/ syllables averaged over left (C3), middle (Cz), and right (C4) central electrodes in the auditory (A), audio-visual (AV), and audio-haptic (AH) modalities. Error bars represent standard errors of the mean. * indicates a significant effect. **Right.** Correlation on raw data between the recognition scores observed in the visual-only and haptic-only modalities in the behavioral experiment (*x*-axis) and the reduction amplitude and latency facilitation observed in the audio-visual and audio-haptic modalities in the EEG experiment (*y*-axis). No correlation was significant.

The main effect of modality was significant [*F*(2,30) = 9.19, *p* < 0.001], with a reduced negative N1 amplitude observed in the AV and AH modalities as compared to the A modality (as shown by *post hoc* analyses, *p* < 0.001 and *p* < 0.02, respectively; on average, A: -5.3 μV, AV: -3.1 μV, AH: -4.1 μV). The interaction between the modality and the syllable was also found to be significant [*F*(4,60) = 7.23, *p* < 0.001]. While for /pa/ a significant amplitude reduction was observed in both AV and AH modalities as compared to the A modality, an amplitude reduction was only observed in the AV modality for /ta/ and /ka/ syllables (as shown by *post hoc* analyses, all *p*’s < 0.001, see **Figure 3A**-left). In sum, these results demonstrate a visually induced amplitude suppression for all syllables and, importantly, an haptically induced amplitude suppression but only for /pa/ syllable.

#### P2 amplitude (see Figures 2 and 3B-left)

No significant effect of the modality [*F*(2,30) = 1.91], the syllable [*F*(2,30) = 1.09] and their interaction [*F*(4,60) = 1.58] was observed.

#### N1 latency (see Figures 2 and 3C-left)

No significant effect of the modality [*F*(2,30) = 0.36], the syllable [*F*(2,30) = 3.13] and their interaction [*F*(4,60) = 1.78] was observed.

#### P2 latency (see Figures 2 and 3D-left)

The main effect of syllable [*F*(2,30) = 4.54, *p* < 0.02] was reliable, with shorter P2 latencies observed for /pa/ and /ta/ syllables as compared to /ka/ (as shown by *post hoc* analyses, all *p*’s < 0.03; on average, /pa/: 210 ms, /ta/: 211 ms, /ka/: 217 ms). Crucially, the main effect of modality was significant [*F*(2,30) = 4.05, *p* < 0.03], with shorter latencies in AV and AH as compared to the A modality (as shown by *post hoc* analyses, all *p*’s < 0.05; on average, A: 223 ms, AV: 208 ms, AH: 207 ms). In sum, these results thus indicate faster processing of the P2 auditory evoked potential for /pa/ and /ka/ syllables. In addition, a latency facilitation was observed in both AV and AH modalities, irrespective of the presented syllables.

#### Correlation between perceptual recognition scores (see Figure 3-right)

For raw data, whatever the modality, no significant correlation was however observed for both N1 amplitude (AV: *r* = 0.09, *p* = 0.54; AH: *r* = 0.06, *p* = 0.70), P2 amplitude (AV: *r* = 0.25, *p* = 0.09; AH: *r* = -0.09, *p* = 0.53), N1 latency (AV: *r* = -0.06, *p* = 0.71; AH: *r* = 0.11, *p* = 0.45), and P2 latency (AV: *r* = 0.07, *p* = 0.66; AH: *r* = -0.01, *p* = 0.92). Results on additional correlation analyses on normalized data also failed to demonstrate any significant correlation for both N1 and P2 amplitude (N1-AV: *r* = 0.01, *p* = 0.98; N1-AH: *r* = 0.18, *p* = 0.87; P2-AV: *r* = 0.21, *p* = 0.15; P2-AH: *r* = 0.02, *p* = 0.91) and latency (N1-AV: *r* = 0.01, *p* = 0.92; N1-AH: *r* = 0.12, *p* = 0.65; P2-AV: *r* = 0.06, *p* = 0.68; P2-AH: *r* = -0.02, *p* = 0.87).

## DISCUSSION

Two main results emerge from the present study. First, in line with our previous results (Treille et al., 2014), a modulation of N1/P2 auditory evoked potentials was observed during live audio-visual and audio-haptic speech perception compared to auditory speech perception. However, contrary to two previous studies of audio-visual speech perception (van Wassenhove et al., 2005; Arnal et al., 2009), no significant correlation was observed between the latency facilitation observed in the bimodal conditions and the degree of visual and haptic recognition of the presented syllables.

Before we discuss these results, it is first important to consider one potential limitation of the present study. Classically, testing cross-modal interactions requires to determine that the observed response in the bimodal condition differ to the sum of those observed in the unimodal conditions (e.g., AV ≠ A + V). However, visual-only and haptic-only modalities were not here tested, due to the technical difficulty to get temporal accurate and reliable triggers for EEG analyses. Notably, because of their temporal limitation and variability, visual and/or surface electromyographic recordings of the experimenter’s lip, jaw or tongue movements would not allowed to determine reliable triggers (especially in the case of lip stretching for /ta/ and /ka/ syllables). From the possibility that the observed bimodal neural responses simply come from a superposition of the unimodal signals, it should however be noted that auditory evoked potentials are rarely observed in the visual-only modality in central electrodes (Besle et al., 2004; van Wassenhove et al., 2005; Pilling, 2010). Furthermore, in our previous study and using the same experimental design, we obtained behavioral evidence for a strong temporal precedence of the haptic and visual signals on the acoustic signal (Treille et al., 2014). In our view, it is therefore unlikely that visual and haptic event-related potentials might arise at the same time-latency and at the same central electrodes that N1 and P2 auditory evoked potentials. For these reasons, we here compared neural responses in each bimodal condition to the related unimodal condition (i.e., AV ≠ A and AH ≠ H), a testing procedure that has previously demonstrated latency facilitation and amplitude reduction of auditory evoked potentials in audio-visual compared to auditory-only speech perception (van Wassenhove et al., 2005; Pilling, 2010).

In spite of this limitation, the observed modulation of N1/P2 auditory evoked potentials in the audio-visual condition strongly suggests cross-modal speech interactions. It is first worthwhile noting that, for each participant, the three syllables were randomly presented in each session in order to minimize repetition effects, and the order of the modality of presentation was fully counterbalanced across participants so that possible overlapping modality effects are unlikely. In addition, auditory-evoked responses were compared between modalities, with the same number of trials and therefore similar possible habituation effects. Although our results appear globally consistent with previous EEG studies, some differences have however to be mentioned. First, while the observed amplitude reduction was here confined to the N1 auditory evoked potential, as in our previous study (Treille et al., 2014; see also Besle et al., 2004), such a visually induced suppression has been previously observed for both N1 and P2 auditory components (Klucharev et al., 2003; van Wassenhove et al., 2005; Stekelenburg and Vroomen, 2007; Pilling, 2010; Baart et al., 2014) or only for the P2 component (Baart et al., 2014). Second, the observed P2 latency facilitation also contrasts with previous studies showing earlier latencies during audio-visual speech perception for both N1 and P2 peaks (van Wassenhove et al., 2005; see also Pilling, 2010, for a small but not consistent effect) or only for N1 peak (Stekelenburg and Vroomen, 2007; Baart et al., 2014; Treille et al., 2014). From these differences, it is hypothesized that N1 and P2 components as well as latency facilitation and amplitude reduction effects might reflect different aspects and/or stages of audio-visual speech integration. For instance, van Wassenhove et al. (2005) observed a visually induced suppression of both N1 and P2 components independently of the visual saliency of the speech stimuli, but a latency reduction of N1 and P2 peaks depending on the degree of their visual predictability. From their results, they argue for two distinct integration stages: (1) a global bimodal perceptual stage, reflected in the amplitude reduction, independent of the featural content of the visual stimulus and possibly reflecting phase-coupling of auditory and visual cortices, and (2) a featural phonetic stage, reflected in the latency facilitation and stronger for P2, in which articulator-specific and predictive visual information are taking into account in auditory phonetic processing (for further discussion, see van Wassenhove, 2013). In parallel, Stekelenburg and Vroomen (2007), Vroomen and Stekelenburg (2010), and Baart et al. (2014) also argue for a bimodal, non-speech specific stage in audio-visual speech integration but here thought to be reflected in the N1 latency facilitation and amplitude reduction. Congruent with this hypothesis, they observed an amplitude and a latency reduction of auditory-evoked N1 responses during audio-visual perception for both speech and non-speech actions, like clapping hands (Stekelenburg and Vroomen, 2007), as well as for artificial audio-visual stimul, like two moving disks predicting a pure tone when colliding with a fixed rectangle (Vroomen and Stekelenburg, 2010). In addition, they also provided evidence for a P2 amplitude reduction specifically dependent on the phonetic predictability of the visual speech input (Baart et al., 2014; see also Vroomen and Stekelenburg, 2010). Taken together, although the observed differences across the present and previous studies on N1 and/or P2 latency facilitation and/or amplitude reduction are still a matter of debate (van Wassenhove et al., 2005; Baart et al., 2014), they might both reflect multistage processes in audio-visual speech integration and also derive from specific experimental settings used in these studies.

From that latter possibility, one interesting finding is that the observed latency and amplitude reduction in the EEG experiment, notably for the P2 component, did not significantly depend on the degree of visual recognition of the speech targets in the behavioral experiment. This contrasts with two previous studies reporting latency shifts of auditory evoked responses directly function of the visemic information (van Wassenhove et al., 2005; Arnal et al., 2009). For instance, van Wassenhove et al. (2005) demonstrated a visually induced facilitation of the P2 auditory evoked potential which systematically varied according to the visual-only recognition of the presented syllable (i.e., the more visually salient was the syllable, the more stronger the latency facilitation). While they observed a P2 latency facilitation around 25 ms, 16 ms, and 8 ms for /pa/, /ta/, and /ka/ syllables, respectively, we here observed latency facilitations around 17 ms, 13 ms, and 15 ms for the same syllables. However, correlation scores likely depend on overall differences in recognition scores between syllables which were stronger in previous studies (van Wassenhove et al., 2005; Arnal et al., 2009). Furthermore, one important difference between our experimental setting and those used in these two studies is that audio-visual interactions were here tested during live face-to-face interactions between a speaker and a listener, with a unique occurrence of the presented syllable in each trial. This natural stimulus variability contrasts with the limited number of tokens used to represent each syllable in the previous studies which were repeatedly presented to the participants (i.e., van Wassenhove et al. (2005): one speaker, three syllables, one token per syllable and 100 trials per syllable and per modality; Arnal et al. (2009): one speaker, five syllables, one token per syllable and 54 trials per syllable and per modality). Similarly, another possible experimental factor impacting bimodal speech integration comes from the number of syllable type. From that view, it is worthwhile noting that we did observe a latency facilitation during live face-to-face speech perception in our previous study, using a similar experimental design, but only for the N1 component (Treille et al., 2014). In this study, however, a simple two-alternative forced-choice identification task between /pa/ and /ta/ syllables was used. It is therefore possible that specific phonetic contents of these two syllables were less perceptually dominant in this previous study, with a more global yes-no strategy done in relation to the more salient bilabial movements for /pa/ as compared to /ta/ (for experimental designs only using two distinct speech stimuli, see also Stekelenburg and Vroomen, 2007; Pilling, 2010; Vroomen and Stekelenburg, 2010; Baart et al., 2014). Overall, given the significant P2 latency facilitation, our results do not contradict the hypothesis that visual inputs convey predictive information with respect to the incoming auditory speech input (for a discussion on the sensory predictability of audio-visual speech stimuli, see Chandrasekaran et al., 2009; Schwartz and Savariaux, 2013) nor the fact that visual predictability of the speech stimulus might be reflected in auditory evoked responses. We simply argue that visual predictions on the incoming acoustic signal in audio-visual speech perception might likely be constrained not only by the featural content of the visual stimuli but also by the experimental context and by short-term memory traces and knowledge the listener previously acquired on these stimuli.

As in the audio-visual condition, the observed modulation of N1/P2 auditory evoked potentials during audio-haptic speech perception also clearly suggests cross-modal speech interactions between the auditory and the haptic signals. In this bimodal condition, we also observed a latency facilitation on the P2 auditory evoked potential that did not vary according to the degree of haptic recognition of the speech targets. In addition to this latency facilitation, an N1 amplitude reduction was also observed but only for /pa/ syllable. As previously noted, this latter result fits well with a stronger haptic saliency of the bilabial rounding movements involved in /pa/ syllable (see Treille et al., 2014, for behavioral evidence) and with previous studies on audio-visual integration demonstrating that N1 suppression is strongly dependent on whether the visual signal reliably predicts the onset of the auditory event (Stekelenburg and Vroomen, 2007; Vroomen and Stekelenburg, 2010). As discussed previously, the fact that P2 latency reduction was nevertheless observed for all syllables indirectly argue for distinct integration processes in the cortical speech processing hierarchy (van Wassenhove et al., 2005; Stekelenburg and Vroomen, 2007; Vroomen and Stekelenburg, 2010; Baart et al., 2014).

Taken together, our results provide new evidence for audio-visual and audio-haptic speech interactions in live dyadic interactions (Treille et al., 2014). The fact that the modulation of N1/P2 auditory evoked potentials were quite similar in these bimodal conditions, despite the less natural haptic modality, further emphasizes the multimodal nature of speech perception. As previously mentioned, apart from speech, multisensory integration from sight, sound and haptic modalities naturally occurs in everyday life. Although bimodal speech perception is a special case of multisensory processing that interfaces with the linguistic system, similar integration processes might have been used to extract temporal and/or phonetic relevant information from the visual and haptic speech signals that, together with the listener’s knowledge of speech production (for a review, see Schwartz et al., 2012), might have constrained the incoming auditory processing.

## Conflict of Interest Statement

The authors declare that the research was conducted in the absence of any commercial or financial relationships that could be construed as a potential conflict of interest.
